# Correlation Between the Chemical Induction of Hyperplasia and of Malignancy in the Bladder Epithelium

**DOI:** 10.1038/bjc.1965.34

**Published:** 1965-06

**Authors:** D. B. Clayson, T. A. Lawson, S. Santana, G. M. Bonser


					
297

CORRELATION BETWEEN THE CHEMICAL INDUCTION OF

HYPERPLASIA AND OF MALIGNANCY IN THE BLADDER
EPITHELIUM

D. B. CLAYSON, T. A. LAWSON, S. SANTANA* AND G. M. BONSER
From the Department of Experimental Pathology and Cancer Research,

The School of Medicine, Leeds, 2

Received for publication December 30, 1964

4-ETHYLSULPHONYLNAPHTHALENE- 1 -SULPHONAMIDE (HPA) induces hyperplasia
of the bladder epithelium in the rat and mouse (Paget, 1958; Sen Gupta, 1962).
The hyperplasia arises within forty-eight hours of the first administration of the
chemical (Bonser and Clayson, 1964) and with repeated dosing is still present
after forty weeks (Sen Gupta, 1962). Carcinomas of the bladder were found in
Ab x IF mice receiving the chemical for up to sixty-five weeks and were more
prevalent in female than in male mice (Bonser and Clayson, 1964).

The discovery of the carcinogenic action of HPA on mouse bladder epithelium
was unusual in so far as the tests were undertaken following observation of the
induction of hyperplasia. Other bladder carcinogens were found, more or less by
chance, as a result of their action over long periods in man or experimental animals
and their early effects have largely been ignored. The present report is an attempt
to remedy this omission.

Relatively few chemicals are known to induce bladder cancer in the experi-
mental animal (Boyland, 1963). In some cases the absence of tumours may have
been due to a failure to examine this organ with sufficient care. Therefore some
hitherto unpublished long term tests in which the bladder has been adequately
examined, are included in this paper in order to strengthen the comparison
between the early effects of a chemical and the formation of tumours.

MATERIALS AND METHODS

Mice of strains CBA and IF, and C57 x IF and Ab x IF Fl hybrids were bred
in the laboratory. Stock albino mice were obtained from a dealer. Stock albino
rats were from the Sheffield colony, Slonaker rats from the Cancer Research
Department of the University of Nottingham and Sprague Dawley rats from a
dealer.

Mice, rats and hamsters were fed on a pelleted diet (Oxo 41B) and water ad
libitmrn. Dogs received one of the commercially available tinned dog foods, hound
meal and water.

Wherever possible, chemicals were obtained commercially. Others were
prepared in the laboratory by standard methods. Dr. A. L. Walpole is thanked
for a gift of 4'-fluoro-4-aminodiphenyl, Dr. B. Silvestrini for a sample of oxo-
lamine (3-phenyl-5/l-diethylaminoethyl-1 2: 4-oxadiazole) and Dr. C. Hackman

* Present address: Hospital das Cliniicas. Universidade da Bahia, Salvador, Bahia, Brazil.

298    D. B. CLAYSON, T. A. LAWSON, S. SANTANA AND G. M. BONSER

for gifts of 2-aminodiphenylene oxide (2-aminodibenzofuran) and 3-methoxv-2-
aminodiphenylene oxide (3-methoxy-2-aminodibenzofurail). Details of the ad-
ministration of the chemicals in the carcinogenicity tests are given in Table I.
Treatment was commenced when the mice were about ten weeks old.

In short term tests chemicals were usually administered by stomach tube under
light ether anaesthesia six times weekly. Details of solvents and dosage are
given in Tables V to IX. In calculating the amount of a chemical consumed in
the diet, it was assumed that a mouse eats approximately 4 g. daily (Tannenbaum.
1942).

The preparation of bladder tissues for microscopical examination has been
described by Sen Gupta (1962). One section from each half of the bisected bladder
of each mouse, rat or hamster was examined for tumours or for hyperplasia.

RESULTS
Carcinogenicity tests

The chemicals used in the carcinogenicity tests differed widely in their toxicity
and this dictated the variation in the total dose administered (Table I). 1-
Phenylazo-2-naphthol was well tolerated at a level of 0.1 per cent in the diet by

TABLE I.- Method of Administration and Amount of (1hemical given in Long-termn

Experiment8

Estimiiated
Type of  Route of                               Duiation  total

Compoundl  mouse administratioin      Concentratioin      (months) dose (mg.)
4-Acetamido- . Ab x IF  Diet    0 03 per cent (intermittently)  1 2    330

stilbene

4-Aminodi-  . Ab x IF  Stornach  0 2 mnl. of 0 25 per cent solutioIn in  9)  38

phenyl               tube       amachis oil. (2 x weekly)

4-Aminostil- . Ab x IF  Diet     0 03 per cent (intermittently)  18   440

bene

4'-Fluoro-4-  CBA    Stomnach    00 5 ml. of 1 per cent in arachis  6   13

aminodi-            tube         oil (2 x weekly)
phenv1

l-Phenylazo- . (BA   Diet        0 1 per cent                  1 2    1400

2-naphthol  stock

both stock and CBA mice. 4-Aminodipheniyl and 4'-fluoro-4-aminodiphenyl were
given by stomach tube because the volatility of the former chemical made it
hazardous to include in the diet and the latter was available only in limited
amounts. It was found possible to give 0-5 mg. of 4-aminodiphenyl twice weekly
for 9 months, but 4'-fluoro-4-aminodiphenyl could only be given in half this
quantity for 6 months.

Ab x IF mice tolerated 0 03 per cent of 4-aminostilbene and 4-acetamido-
stilbene in the diet for about 20 weeks. The mice then developed paralvsis of the
hind legs and became progressively more emaciated. Partial recovery occurred
when the animals were fed on the basal diet for one month and thereafter the
chemical and basal diets were alternated weekly. The mice retained their
emaciated appearance and often developed coineal opacities. It was suspected
that the symptoms might be the result of peripheral neuritis, but histological
examination failed to confirm this.

EFFECT OF CHEMICALS ON BLADDER EPITHELIUM

Morphological changes in the bladder epithelium

Bladders were examined histologically in mice surviving for 50 or more weeks
in the following instances  Ab x IF controls (38 mice); 4-aminodiphenyl (15
mice); 4-aminostilbene (23 mice); 4-acetamidostilbene (32 mice); 4'-fluoro-4-
aminodiphenyl (20 mice) and 1-phenylazo-2-naphthol (28 mice).

Hyperplastic bladder epithelium, considered to be unrelated to the treatment,
was occasionally observed in untreated Ab x IF mice and in those fed 4-amino-
stilbene or 4-acetamidostilbene. With 4-aminodiphenyl one male Ab x IF mouse
at 80 weeks and four at 98 weeks had hyperplastic bladder epithelium. A male
mouse at 96 weeks appeared on naked eye examination to have a tumour of the
bladder and a female mouse at 97 weeks and a male at 98 weeks had Grade II
carcinomas (i.e. the tumours were invading the muscular wall). The induction of
two confirmed carcinomas of the bladder in 12 mice surviving to 90 weeks is
suggestive, but not conclusive evidence of carcinogenic activity.

Tu7mours of the liver

Livers of Ab x IF mice developed hepatomas without treatment (Table II).
The incidence was 40 per cent in males and 14 per cent in females and was not
significantly affected by administration of 4-aminodiphenyl. 4-Aminostilbene
and 4-acetamidostilbene, however, depressed the incidence of hepatomas in
males to 9 and 6 per cent, respectively, and in females to zero.

The untreated CBA mice include those described by Williams and Bonser
(1962). The additional 15 male and 18 female mice(TableIII)havenotappreciably
altered the overall incidence of hepatomas. Feeding I-phenylazo-2-naphthol in
the diet did not materially affect the incidence of hepatomas, but the limited dose
of 4'-fluoro-4-aminodiphenyl (13 mg.) raised the incidence from 12 to 49 per cent
in males and from 4 to 63 per cent in females. In 3 of the male mice cholangiomas
as well as hepatomas were present. Centrilobular necrosis was found in 4 of 6
female mice killed between 16 and 30 weeks and in another at 83 weeks.

4'-Fluoro-4-aminodiphenyl was also administered to 50 male and 31 female IF
mice. Only 13 males and 3 females survived to between 50 and 80 weeks. The 3
hepatomas observed (one in a male at 62 weeks and two in females at 54 weeks)
are probably significant because no hepatomas were seen at post mortem in 63
male and 133 female breeding IF mice surviving from 50 to 111 weeks. From the
above data 4'-fluoro-4-aminodiphenyl is judged to be carcinogenic in CBA and IF
mice.

The untreated stock mice are those described by Clayson and Ashton (1963).
The addition of I -phenylazo-2-naphthol to the diet slightly depressed the incidence
of hepatomas inl the males.

Intestinal tumours

The ileo-caecal junction was examined histologically in the majority of mice
as tumours were found at this site in earlier work (Bonser, Clayson and Jull, 1956).
One benign caecal papilloma was found in an Ab x IF mouse which had been
treated with 4-aminostilbene for 104 weeks.

The untreated stock mice developed caecal adenomas, usually associated with
cysts, in 7 out of 49 instances and one male mouse had a carcinoma in this region.

Pe) Q

300   D. B. CLAYSON, T. A. LAWSON, S. SANTANA AND G. M. BONSER

r-c0   c 10    r-     -c
0101  -4      --   _

0  0 o
01-

01 *  .NO

I1I     I        11

I C1  S_ -q

II       -z

1 1

1
-0o

01  1 .1~   0 n .
0   00  00 o 0

0D
01  0101  01

00  00  0~~~~~~C
tl-  t1 t1  eKl 0

._

*  ~     ~~~~~~~ 0

-~~~~ 0   0

?   0 ? 0 ? ? C

a)  0

=0  0   0

0     ce

?~~       H

* );

0

* )
* )2

*0;
0)

0
0)v

* )a

1.

H

H 10

O --
t-  ro0

cs e  E

O o _

0
g

0.
0e :

= Oq

o  0) I  ]t
0

a) W

ce0?I   1

o.  0

0

00Fi0

._

V

0
05

z

oo 0
0-1

1
-  O

--    >t

X*   0C

~ ~1
-- 00

.    .   *   Sq

_     0:
<    C   0

*       "0C1

9 0 0

._e 0

0   "-    -

01;   0    -

.H O~ C;

S:    -

C     IA      -     o      in                I

F )

-? Z "o

0 0  C) ^

C,)

H-

EH

0     1

.

D 01_

s0 (D

0~    -
.0010I
X

C

0 0f

00 C)

0qS

K

.0 X lo

S7 L

1-0 C-4

' -e
-.

*0C

xs

-0
*0i

0
*HS

*

1 -4

01 0 1-O0

I I

_ I

- I

-.

.0

C;

._

C)

0

z

V

cI-

EFFECT OF CHEMICALS ON BLADDER EPITHELIUM

Stock mice treated with I-phenylazo-2-naphthol developed adenomas, often
associated with cysts, in 6 out of 23 cases.
Tumour8 of other tis8ues

These were apparently unrelated to the treatment. Four untreated female
Ab x IF mice had breast carcinomas, one had a probable sarcoma and one a
leiomyosarcoma of the cervix, one a myoma and one a haemangiomatous polyp of
the uterus, 4 had cystic ovaries and one a granulosa cell tumour. In the male
there were two certain and one probable sarcomas (of the epididymis, the shoulder
and the back). There were also two papillomas of the stomach and two instances
of leukaemia, one of each being in a female and the other in a male. As would be
expected with an A strain parent, lung adenomas were frequent.

Similar tumours were observed distributed at random in the chemically treated
Ab x IF mice. The only features of interest were one renal carcinoma in a male
90 weeks after the start of treatment with 4-acetamidostilbene and four granulosa
cell tumours of the ovary 89 to 104 weeks after the start of treatment with 4-
aminostilbene.

CBA mice were relatively free from spontaneous tumours other than of the
liver. Both the control and the chemically treated groups developed a low in-
cidence of granulosa cell tumours of the ovary. There were two reticulum cell
tumours of the ovary in the group treated with I-phenylazo-2-naphthol and a
probable similar tumour with 4'-fluoro-4-aminodiphenyl. Five mice receiving
I-phenylazo-2-naphthol had leukaemia.

The female stock mice used developed mammary carcinomas, and ovarian
cysts and tumours, without chemical treatment.

TABLE IV.-The Incidence of Hepatomras in Stock Mice With and Without

Chemical Treatment

Number of hepatomas in mice
dying within stated period of
weeks after birth (controls) or

start of treatment

- ,Al                   'N            Incidence
Compound        Sex     50-59 60-69 70-79 80-89 90-99  Total  (per cent)

None      .        .  .        -   1/6  3/8  0/4   -    . 4/18   .    22

F.   . 0/1   0/3  0/11 0/14 0/2   . 0/31   .    0
1-Phenylazo-2-naphthol  AM.  . 0/3  0/2  1/4  0/7       . 1/16   .     6

F.   .       0/2  0/6  0*/11      . 0/19   .    0
* One mouse with reticulum cell sarcoma.

Short Term Tests

The results of the short term tests are summarised in Tables V to IX. In
considering these results it was decided to use a numerical formulation in order to
assess the fine differences which occur in individual bladders. Five points were
scored for a hyperplastic bladder epithelium in which there were foci, large areas or
the whole of the epithelium composed of six to eight cell layers; three points
were scored for a mildly hyperplastic bladder epithelium with three to five cell
layers, and one point for doubtful hyperplasia, that is to say for abladderepithelium
which was not normal but had not progressed to the mildly hyperplastic stage.

301

302   D. B. CLAYSON, T. A. LAWSON, S. SANTANA AND G. M. BONSER

The " Hyperplastic Index " was defined as 20ON/n where N is the total number of
points scored in a group of animals and n is the number of animals in the group.
This index can have a range of values from 0 to 100 and as a result of our experience
we suggest that when a section from each half of the bisected bladder is examined,
an index of more than 50 indicates that the administered chemical induces hyper-
plasia, an index of under 30 indicates inactivity, while the interim between 30 and
50 represents an indecisive result. The latter category was infrequent.

4-Ethylsulphonylnaphthalene- 1 -sulphonamide (HPA) and 2-acetamidofluo-
rene are the only unequivocal bladder carcinogens in the mouse (Armstrong and
Bonser, 1947; Bonser and Clayson, 1964). Both compounds have high hyper-
plastic indices whether given by stomach tube or fed in the diet (Table Vr).
4-Aminodiphenyl has been shown to induce two bladder carcinomas in 12 surviving
mice after a long latent period and is thus probably a bladder carcinogen in this
species. It has hyperplastic activity when given by stomach tube but not when
incorporated in the diet.

Large numbers of chemicals have been tested for carcinogenic acitivity in the
mouse and cancer of the bladder has not been reported. In order to obviate the
possibility of the inclusion of bladder carcinogens among compounds thought not
to induce cancer of the bladder, it was decided to use only those compounds tested
in Leeds where the bladders were examined to establish the absence of cancer.
2-Naphthylamine (Bonser, Clayson, Jull and Pyrah, 1956), benzidine, 1-methoxy-
2-naphthylamine (Bonser, Clayson and Jull, 1956), auramine (Williams and Bonser,
1962), 1-naphthylamine (Clayson and Ashton, 1963), 4-aminostilbene (this paper)
and o-aminoazotoluene (Bonser and Clayson, unpublished results) are not carcino-
genic to the bladder and with the exception of o-aminoazotoluene have low
hyperplastic indices (Table VI). The latter chemical had an index of 42 when
given at a level of 1 mg. daily. The toxic nature of this treatment led to the death
of all the animals by 6 weeks. Administration of the chemical on alternate days
reduced the hyperplastic index to 31, a value indicating that o-aminoazotoluene is
probably not without some degree of hyperplastic activity in the bladder epi-
thelium. When the chemical was given in the diet rather than by stomach tube
much less hyperplastic activity was exhibited, a result which parallels that
obtained with 4-aminodiphenyl (Table V).

It was next decided to investigate a group of chemicals of which the carcino-
genic activity in the mouse bladder is not known (Table VII). The first three of
these induce cancer of the bladder in the rat. 2-Aminodiphenylene oxide is a
weak and 3-methoxy-2-aminodiphenylene oxide a more potent bladder carcinogen
in the rat (Hackmann, 1959). The former has a high hyperplastic index in the
C57 x IF mouse and the latter an equivocal index. Di-n-butylnitrosamine
induced tumours of the bladder in six of sixteen rats (Druckrey, Preussmann,
Schmahl and Muller, 1962). It induces hyperplasia in the mouse. Oxolamine
(3-phenyl-5,8-diethylaminoethyl- 1: 2: 4-oxadiazole) induced hyperplasia of the
bladder when given in high doses to the dog and rat but not to the mouse (Silves-
trini, Bignami, Garan and Pozzatti, 1963). It has also been claimed to induce
bladder tumours in the two former species (Barron, 1963).* In the present experi-

* One of us (G. M. B.) has seen Dr. Barron's material and is unable to agree that he has obtained
frank carciinomas of the bladder by the administration of oxolamine. The lesions in the dog and the
rat appear to be precancerous and might have progressed to frank carcinoma if the animals had been
permitted to live for more than 12 months.

EFFECT OF CHEMICALS ON BLADDER EPITHELIUM

C)

4

._

oso

*Q,

u r-

CD

0

52-       "   :    I      I

0         t1

X       w     U0I        I

)    v

5

C)?          X     n
o         ?

CO            >
~ o

-     C

C)               4

> ? o~~

I ')

Eq       v  >,,=_~~~~~C

O    N00 t   C0  OM

X  o r o  c

C)

S I !I  I  I  I

01~~~~~~~

4z._
t1 I I  II I  I

C).-

I I  I  I  It  I   C

N   --         C,

CX    ~~~04

I  I  I II   I  C)

-~ ~~~ -

O  -  I--  I  I -

10  10  10  10t  10  10< 1  0

o  0   0 0   0 V   o

.E

-     o

*

0     0    0

C)               C
-~~~~ W

O

*    C);

4                 ?

01

303

tl?

304   D. B. CLAYSON, T. A. LAWSON, S. SANTANA AND G. M. BONSER

o       100        010    0 (

P-                  r-

C     -    0 o        V0     co
C-          C1        -4

02

0

010

0

I         -~~~~~~~4

I  I  III   I   I~~-I  I  I  +~    .-V

m   cli m     N C    w l
O      o     (=   (= '.. 0   o

I           I           I        I

0

O         C)      (:     "  -  -4     C    +

01  01 ~~~~ CO

CO   01 I O 0  I0O    W

0     00    0  -  0   -l

X    X X   X XX   X   X

o       0

.a2   .      .t   U2

^   . _  ^  ^   _   .

C.    ^  )  ,s  _)

I     I    I

01~

I   II   I

- -

+ X

t-     E-
10    410

0      0

_++

4---

*-  -.,

0

0~~~~~~~~~~

V S       D~~~~ S 3

.d ~~             0

?,   =  t   t  ?~~~~~

t    m   W~0  -  0

?t_

V1

0

0
o.

- .

o

*d

0      0

. z

* *5 l1i1

.  H

.d    o

0

4-'a
m

03 x
1-4

$:L, Q

;. t? II*
0 0

?14 "

?i

*
0O

B

0
0

._

0

0

0
0

._

(D
C)

as

ib

t-   I

CO  -

0

1 N
O

L.       I

4o
Co

2

*C5

C.)

C.)
* zQ

IY
?q

( D Hi
0 0 V

0-    -

H bD

d

1b1

0   0
t   . t

._

?2  a

r= I

0

-4

EFFECT OF CHEMICALS ON BLADDER EPITHELIUM

pt    C)

e *

rV  *   X  I   I   ~ ~ ~~~~~~~~~~~~~i  I

D

0)

oo

x:     ~I    t      '0I

tC.  5~  -X.   R

G co_ H.    'I        Io
Zs           0o

ez ~ ~ ~ ~ 0

0        -Q

~~~~~, ~~~~~~en

0~~~

OW

0
o

<    a         |X~~~

C;,       0

0 )                                                0 a                     0=              0O

to                                                P-(                   r-

:   I   I  "I     I

I    I    I   I    I

0)      CS1

I           I         I
I       I

*xX  l <  I               m                   :~ I I                         m I

n           I           I          I        I             I         o                I              I

I   0   0      0  10 c  N

o>  o  P-   o   o

0)

0

S   -  0'1  C n 0)) C   C 0)

-t  0 m  I  t1e 10  0 0 0

-I~~~~~~~~~~-

+lc)  j  a1 t 0. )C  I  I

-  ?   )  -   -  01

~  0  4)

O  ?  O

0 *     . *

0)

-

CO    D

0)
o     S

0

Z     .

~ 8~

*OD-

0

0                     0~~~~~~

.  .*.  .   *  .   *   .

*   *   *   A   *~~0  *  *  iii

_~     ~~ a

13

305

306   D. B. CLAYSON, T. A. LAWSON, S. SANTANA AND G. M. BONSER

ments the absence of any hyperplastic effect in the urinary tract epithelium of the
mouse has been confirmed.

5-Chloro-o-toluidine is responsible for inflammation and haematuria among
workmen handling it in the chemical industry. It did not show a hyperplastic
action in the mouse bladder when fed by stomach tube at a level of 1 mg. per day.
4-Dimethylaminoazobenzene is a weak hepatocarcinogen but has not been
reported to induce bladder cancer in the mouse, although tumours of this site are
evoked in the dog (Nelson and Woodard, 1953). It is without short term action
on the mouse bladder. 3-Hydroxyanthranilic acid has been shown to induce
carcinomas of the bladder epithelium when implanted into the lumen in cholesterol
pellets (Allen, Boyland, Dukes, Horning and Watson, 1957; Clayson, Jull and
Bonser, 1958; Bryan, Brown and Price, 1964). It is without hyperplastic activity
when given systemically to the mouse. 20-Methylcholanthrene is not known to
induce bladder cancer following systemic administration and has no hyperplastic
action.

Finally it was decided to examine the reaction of species other than the mouse.
The rat, hamster and dog have responded to the administration of chemicals by
developing tumours of the bladder.

A group of Sheffield rats treated with 2-acetamidofluorene for 12 days failed
to show any hyperplastic activity (Table VIII). This accords with the failure
of 2-acetamidofluorene to induce bladder cancer in this colony (Bielschowsky,
1944). The use of Slonaker rats of which the bladders are susceptible to the
chemical (Wilson, DeEds and Cox, 1941) led to a hyperplastic index of 72. No
hyperplasia was observed in the bladders of rats killed up to 4 weeks (index = 0)
but all of those killed at 5 and 8 weeks had mild hyperplasia (index- 60). o-Amino-
azotoluene produced a hyperplastic index of 53 in Sprague-Dawley rats. Bladder
cancer was reported in rats treated with this chemical by Yoshida (1935) but his
observation has not been confirmed in other laboratories.

The hamster (Table IX) develops bladder tumours under the influence of
o-aminoazotoluene (Tomatis, Della Porta and Shubik, 1961), but not under that of
2-acetamidofluorene (Della Porta, Shubik and Scortecci, 1959). Hyperplasia
was induced by both chemicals in every animal tested. On the other hand,
4-ethylsulphonylnaphthalene-1-sulphonamide (HPA) of which the carcinogenicity
to this species is not known, did not induce as marked hyperplasia as either of the
foregoing chemicals.

2-Naphthylamine is a potent bladder carcinogen in the dog (Bonser, Clayson,
Jull and Pyrah, 1956). When the chemical (400 mg. daily) was administered to a
dog for 2 weeks, extensive epithelial hyperplasia of the bladder was induced. In a
second dog treated for 4 weeks there was mild patchy hyperplasia. Benzidine is
at the most only weakly carcinogenic to the bladder of the dog (Bonser, 1962,
1963). When this chemical was fed to the dog (300 mg. daily) for 2 or 4 weeks, no
hyperplasia of the bladder epithelium was observed.

There were changes in the ureters and kidneys of these dogs. The dog treated
with 2-naphthylamine for 2 weeks had a unilateral hydroureter with a mild
ureteritis but without any accompanying hyperplasia. At the point where this
ureter passed through the bladder wall there was hyperplasia up to eight cell layers
thick. The other 2-naphthylamine-treated animal showed mild inflammation at
the top end of the ureter. The dog treated for 2 weeks with benzidine showed
unilateral hydronephrosis and epithelial hyperplasia in the lower third of the

EFFECT OF CHEMICALS ON BLADDER EPITHELIUM

TABLE VIII.-Incidence of Hyperplasia of the Bladder Epithelium in Rats Treated with

Chemicals for Short Periods

Hyperplasia in rats killed after

stated period of weeks

Method of               Type of                                I- Hyperplastic
Coolipoun(l  administration  Dose      rat       Sex   1     2    4     5    8      ind(lex
2-Acetamido-  Stomach tube 7 mg /day  Sheffield    M.    -  0*/12   -*

fluorene

fluore.ne   Stomach tube 3 mg./day   Slonaker   M+F. 0/2    0/1  0/2   2M    2M       27

o-Aminoazo-   Diet           0- 1     Sprague-     AL                   9 + 21M  IM     53

toluene                  percent      Dawley                            3     3

* These rats had bladder pouches and the degree of hyperplasia did not differ from that in controls.

TABLE IX. Incidence of Hyperplasia of the Bladder Epitheliumr in Hamrsters treated with

various Chemicals for Short Periods

Carcinogenic
activitv to
Method of  Duration   Dose                           Hyperplastic  bladder

Comiipounid  administration  (weeks)  (per cent) Sex Degree of hyperplasia  index  epitheliurn
HPA    .             Diet        10   . 0-01    M. .  Mild hyperplasia (2/3).  40  . Not known
o-Aininoazo-         Diet         6   . 0-1    . M. . Marked in 2, mild in 1  87  . P3itive

toluene

2-Acetarnido-        Diet         6     0-05   . F. . Marked in 2, mild in 2  80  . Negttive

fluiorene

ureter.  The epithelium   at the ureteric orifice was not hyperplastic.    These
changes in the kidney and ureter are thought to be due to the chemical treatment.

DISCUSSION

Of five chemicals tested for carcinogenic activity in the mouse, 4-aminodiphenyl
induced carcinomas of the bladder in low yield and with a long latent period. The
total amount of chemical administered was small because of its toxicity, and in the
hope of obtaining a more substantial result the experiment is being repeated in a
different strain of mouse. The only other carcinogen to be demonstrated was
4'-fluoro-4-aminodiphenyl which induced hepatomas in CBA and in IF mice.

The failure of l-phenylazo-2-naphthol to augment the incidence of hepatomas
in CBA or stock mice was disappointing as Kirby and Peacock (1949) found that
this chemical induced liver cancer in their mice. Two possibilities may explain
the discrepancy. The experimental conditions were different, as Kirby and Pea-
cock injected the chemical into their stock mice whereas in the Leeds experiment
it was fed to another type of stock mouse or to CBA mice. Alternatively, Kirby
and Peacock's control group may have been inadequate as the chemically treated
mice developed hepatomas when they were 18 to 21 months old (15 to 18 months
after the start of treatment) whereas the controls were stated to be " more than 14
months old ".

4-Aminostilbene and 4-acetamidostilbene depressed the incidence of hepatomas
in Ab x IF mice. Both chemicals were toxic at the dose given and the animals

307

308   D. B. CLAYSON, T. A. LAWSON, S. SANTANA AND G. M. BONSER

became emaciated, had hindleg paralysis and later in the experiment developed
corneal opacities. Tannenbaum and Silverstone (1949) showed that both under-
feeding and caloric restriction, which led to reduced body weight, depressed the
incidence of spontaneous hepatomas in male C3H mice and it is probable that the
diminution in the yield of spontaneous hepatomas in mice treated with the stilbene
derivatives is also associated with reduced body weight.

The carcinogenicity tests extend the number of chemicals for which there is
reasonable certainty of failure to induce bladder cancer in the mouse. They and
other compounds tested for carcinogenic activity, mainly in Leeds, form the basis
of the investigation into whether or not, after the systemic administration of a
chemical, the induction of early hyperplasia and the ultimate development of
carcinomas of the bladder epithelium are related. Despite the paucity of available
bladder carcinogens the results in the mouse, rat, hamster and dog support such a
correlation. Some of the chemicals of unknown carcinogenic activity which have
been shown to induce early hyperplasia in the epithelium of the mouse bladder will
therefore be tested for the former property.

The most important apparent discrepancy is the failure of the Slonaker rat to
develop marked hyperplasia after the administration of 2-acetamidofluorene, a
known bladder carcinogen in these animals. It was first suspected that the
Slonaker rats which had been employed were not related to those used by Wilson
et al. (1941) in their early work on the toxicity of 2-acetamidofluorene. These
workers derived their rats from the colony maintained by Stanford University,
California, in 1931. The British Slonaker rats are descended from those sent by
Stanford University to Dr. Kenneth Baker in 1951 or 1952. He, in turn, passed
them to Dr. A. L. Walpole of Imperial Chemical Industries Ltd. (Pharmaceuticals
Division) who in February, 1956 sent a breeding nucleus to the Cancer Research
Department of the University of Nottingham who have inbred them since then.
The experiment described in this paper was performed on animals from the latter
source. So far as can be ascertained the carcinogenicity of 2-acetamidofluorene
has not been assayed on the Slonaker rats in use in this country. However, their
bladder epithelium was more sensitive to the carcinogenic action of 3: 2'-dimethyl-
4-aminodiphenyl than that of the Wistar rat (Walpole, Williams and Roberts, 1955)
and without direct evidence it would be unwise to assume that Nottingham-bred
Slonaker rats are resistant to the induction of bladder tumours by 2-acetamido-
fluorene.

In view of the development of mild hyperplasia in the 2-acetamidofluorene-
treated Slonaker rats killed after 5 and 8 weeks, a more plausible explanation of
the discrepancy is to be found in the increasing amounts of the suspected carcino-
genic metabolite of 2-acetamidofluorene, the N-hydroxy derivative, during con-
tinued dosing. Cramer, Miller and Miller (1960) showed that the amount of the
N-hydroxy derivative in the urine increased from a low level to about 10 per cent
of the administered dose of acetamide during the first 6 weeks of treatment. Thus,
if N-hydroxylation is an essential step in the induction of hyperplasia as well as of
bladder cancer, the delay in the appearance of hyperplasia can be explained. This
speculation needs experimental investigation.

Some compounds have been found which induce early hyperplasia but are not
known to be bladder carcinogens. o-Aminoazotoluene, for example, induces
slight hyperplasia in the mouse and 2-acetamidofluorene hyperplasia in the
hamster, but in neither case do the compounds induce cancer of the bladder. In

EFFECT OF CHEMICALS ON BLADDER EPITHELIUM

these instances the chemicals cause tumours at other sites and it is possible that the
animals do not survive to develop bladder tumours.

Is long continued hyperplasia the only epithelial change necessary for the
induction of cancer of the bladder? In the present experiments most of the chemi-
cals have the ability to induce cancer in one or more tissues and therefore may apply
a generalised carcinogenic stimulus throughout the body. Such a stimulus is most
likely to manifest itself as a cancer in situations where there is an excessive
proliferation of cells, i.e. hyperplasia. Experience in other tissues (Berenblum,
1944) indicates that there are factors in carcinogenesis other than hyperplasia.

Oxolamine (Silvestrini et al., 1963) induced hyperplasia of the bladder in the
rat when large single daily doses were given but not when the same dose was
administered in portions during the day. Hyperplasia appeared to depend on
attaining a peak concentration of the active agent, thought by the Italian workers
to be diethylamine. Two similar examples have been found in the present
experiments: 4-aminodiphenyl and o-aminoazotoluene induced hyperplasia when
given to the mouse in single doses by stomach tube, but not when an equivalent
dose was spread out by administration in the diet. It would help in deciding the
importance of hyperplasia in the induction of cancer if the incidence of bladder
tumours could be compared after the administration of 4-aminodiphenyl in the
diet and by stomach tube.

The chemical and food industries are faced with the prospect oftesting chemicals,
which are to be used in the human environment, for carcinogenic activity. This
is both time consuming and expensive. It is pertinent to ask whether the estab-
lishment of a correlation between early hyperplasia and ultimate malignancy of the
bladder epithelium would in any way help in eliminating potentially dangerous
compounds. From the work which has been described, it appears that there is a
fair degree of correlation between the two processes. It would therefore seem
feasible to argue that certain chemicals are likely to induce cancer of the bladder
on the basis of preliminary toxicity studies. If similar early changes were to be
established in other tissues, and this calls for much more experimental work, it is
conceivable that industry would be able to regard chemicals inducing these early
changes as potentially dangerous and to reject them. It would then be possible
to concentrate long term carcinogenicity tests on fewer compounds which could be
investigated more thoroughly.

SUMMARY

1. 4'-Fluoro-4-aminodiphenyl induced hepatomas in CBA and IF mice.

2. 4-Aminodiphenyl induced two confirmed carcinomas of the bladder in twelve
mice. It is regarded as probably carcinogenic to the mouse.

3. l-Phenylazo-2-naphthol, 4-acetamidostilbene and 4-aminostilbene were not
carcinogenic but the two latter chemicals depressed the incidence of spontaneous
hepatomas in Ab x IF mice. This is thought to be associated with the toxicity
of these substances.

4. The ability of a number of chemicals to induce early hyperplasia of the
bladder epithelium has been investigated in the mouse, rat, hamster and dog.

5. It is considered that there is a reasonable degree of correlation between
early hyperplasia and the ultimate development of malignancy.

6. These results are discussed in relation to the part played by early hyperplasia

3093

310   D. B. CLAYSON, T. A. LAWSON, S. SANTANA AND G. M. BONSER

in the carcinogenic process and to the bearing it may have on the detection of
potential environmental carcinogens.

REFERENCES

ALLEN, M. J., BOYLAND, E., DUKES, C. E., HORNING, E. S. AND WATSON, J. G. (1957)

Brit. J. Cancer, 11, 212.

ARMSTRONG, E. C. AND BONSER, G. M.-(1947) J. Path. Bact., 59, 19.
BARRON, C. N.-(1963) Exp. molec. Path., Suppl. 2, 1.

BERENBLUM, I.-(1944) Arch. Path. (Lab. Med.), 38, 233.
BIELSCHOWSKY, F.-(1944) Brit. J. exp. Path., 25, 1.

BONSER, G. M.-(1962) Acta Un. int. Cancr., 18, 538.-(1963) Brit. Emp. Cancer Campgn,

41, 467.

IdeM AND CLAYSON, D. B.-(1964) Brit. J. Urol., 36, 26.
Iidem AND JULL, J. W.-(1956) Brit. J. Cancer, 10, 653.
Iidem AND PYRAH, L. N.-(1956) Ibid., 10, 533.

BOYLAND, E.-(1963) 'Biochemistry of Bladder Cancer'. Springfield (Thomas).
BRYAN, G. T., BROWN, R. R. AND PRICE, J. M. (1964) Cancer Res., 24, 596.
CLAYSON, D. B. AND ASHTON, M. J.-(1963) Acta Un. int. Cancr., 19, 539.
Idem, JULL, J. W. AND BONSER, G. M.-(1958) Brit. J. Cancer, 12, 222.

CRAMER, J. W., MILLER, J. A. AND MILLER, E. C. (1960) J. biol. Chem., 235, 885.

DELLA PORTA, G., SHUBIK, P. AND SCORTECCI, V.-(1959) J. nat. Cancer Inst., 22, 463.

DRUCKREY, H., PREUSSMANN, R., SCHMXHL, D. AND MULLER, M.-(1962) Naturwissen-

schaften, 49, 19.

HACKMANN, 0. (1959) CIBA Foundation Symposium ' Carcinogenesis: Mechanisms of

Action', p. 308. Edited by G. E. W. Wolstenholme and M. O'Connor. London
(Churchill).

KIRBY, A. H. M. AND PEACOCK, P. R. (1949) Glasg. med. J., 30, 364.

NELSON, A. A. AND WOODARD, G. (1953) J. nat. Cancer Inst., 10, 1205.

PAGET, G. E. (1958) 'A Symposium on the Evaluation of Drug Toxicity'. Edited by

A. L. Walpole and A. Spinks. London (Churchill).
SEN GUPTA, K. P.-(1962) Brit. J. Cancer, 16, 110.

SILVESTRINI, B., BIGNAMI, A., GARAN, A. AND POZZATTI, C. (1963) Exp. molec. Path.,

Suppl. 2, 50.

TANNENBAUM, A.-(1942) Cancer Res., 2, 460.

IdeM AND SILVERSTONE, H. (1949) Ibid., 9, 724.

TOMATIS, L., DELLA PORTA, G. AND SHUBIK, P. (1961) Ibid., 21, 1513.

WALPOLE, A. L., WILLIAMS, M. H. C. AND ROBERTS, D. C.-(1955) Brit. J. Cancer, 9, 170.
WILLIAMS, M. H. C. AND BONSER, G. M. (1962) Ibid., 16, 87.

WILSON, R. H., DEEDS, F. AND Cox, A. J.- (1941) Cancer Res., 1. 959.
YOSHIDA, T. (1935) Gann, 29, 295.

				


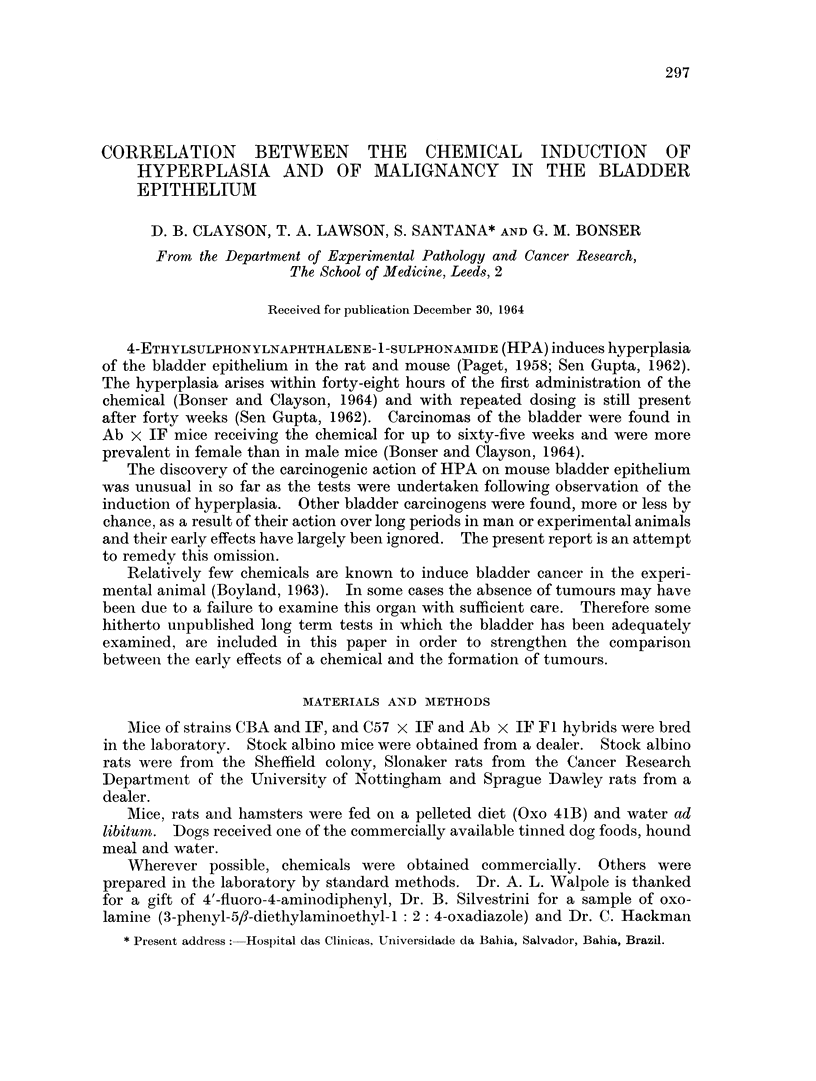

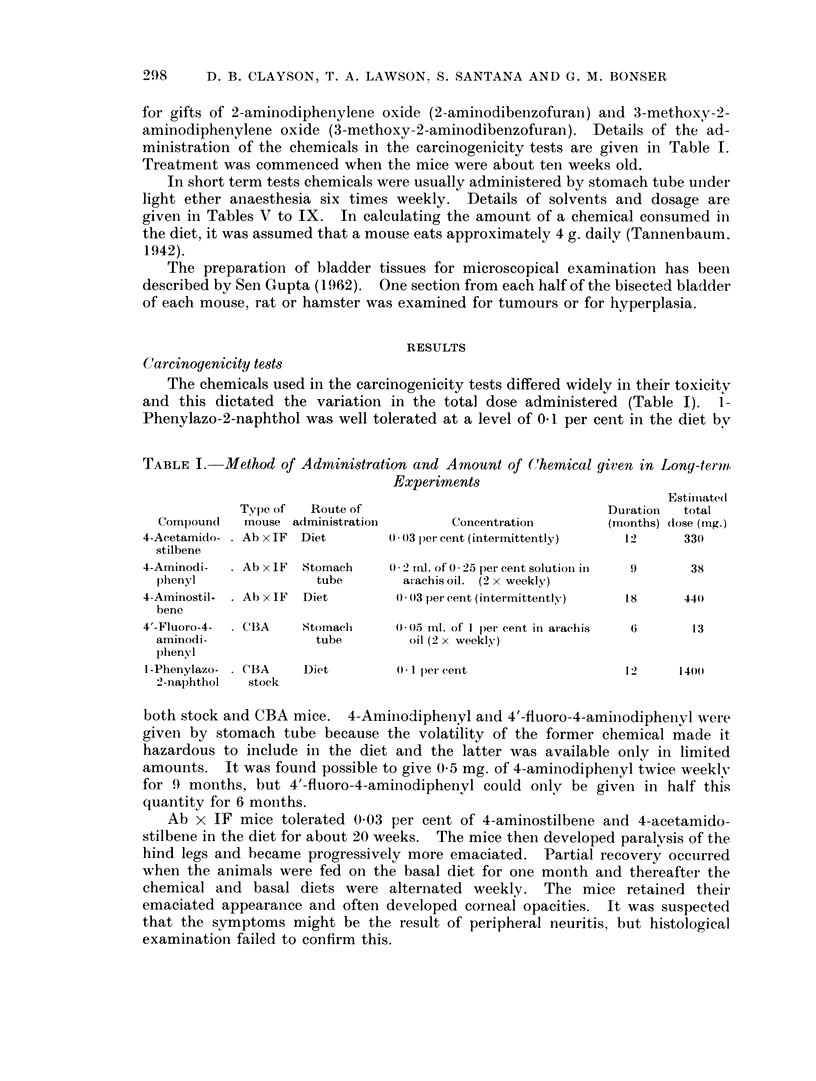

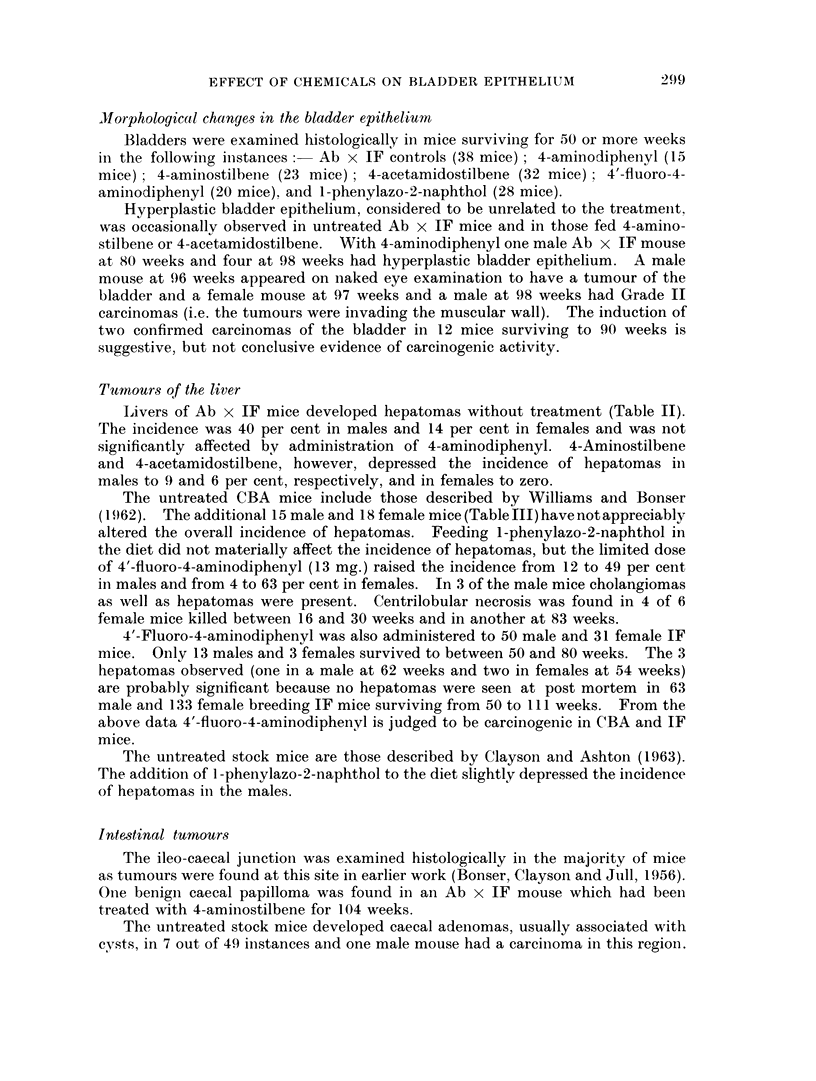

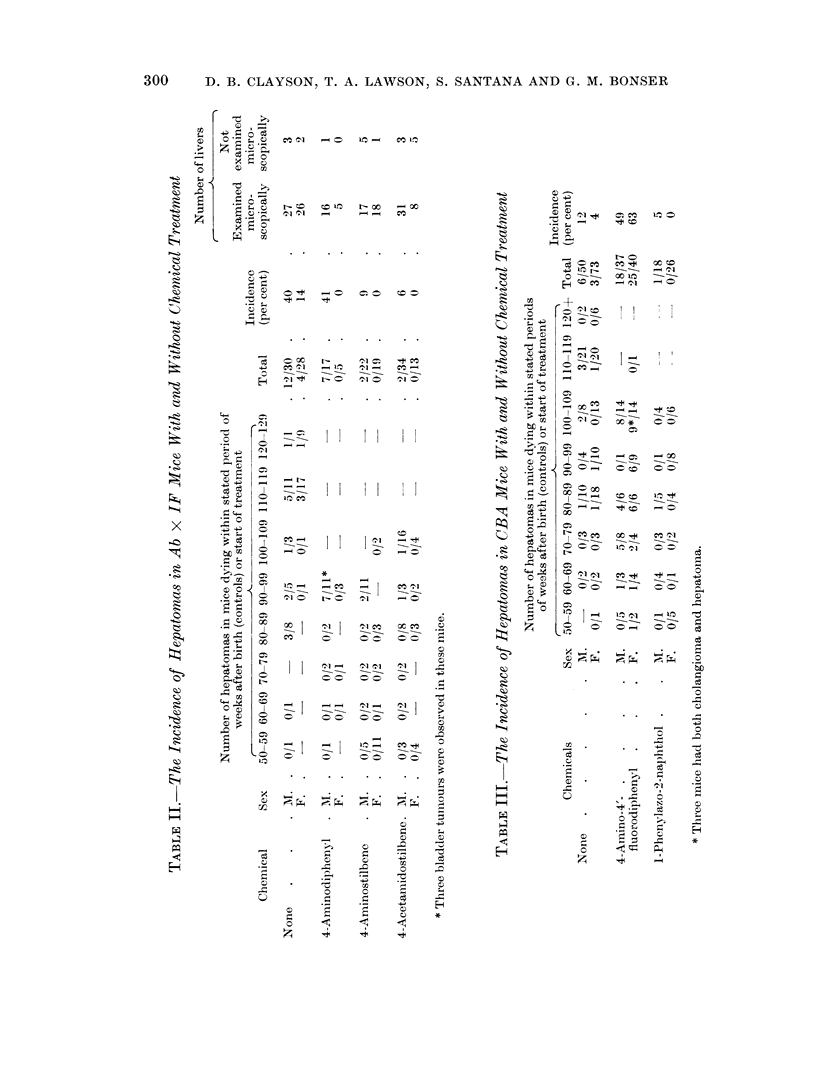

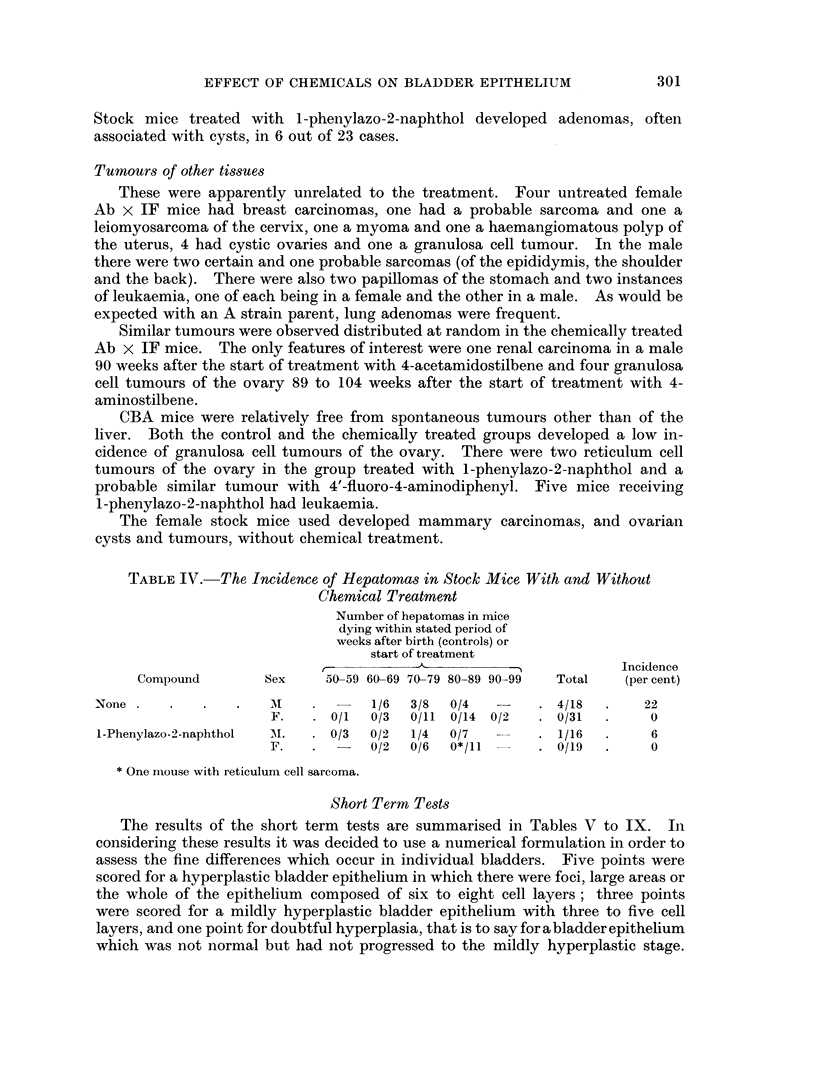

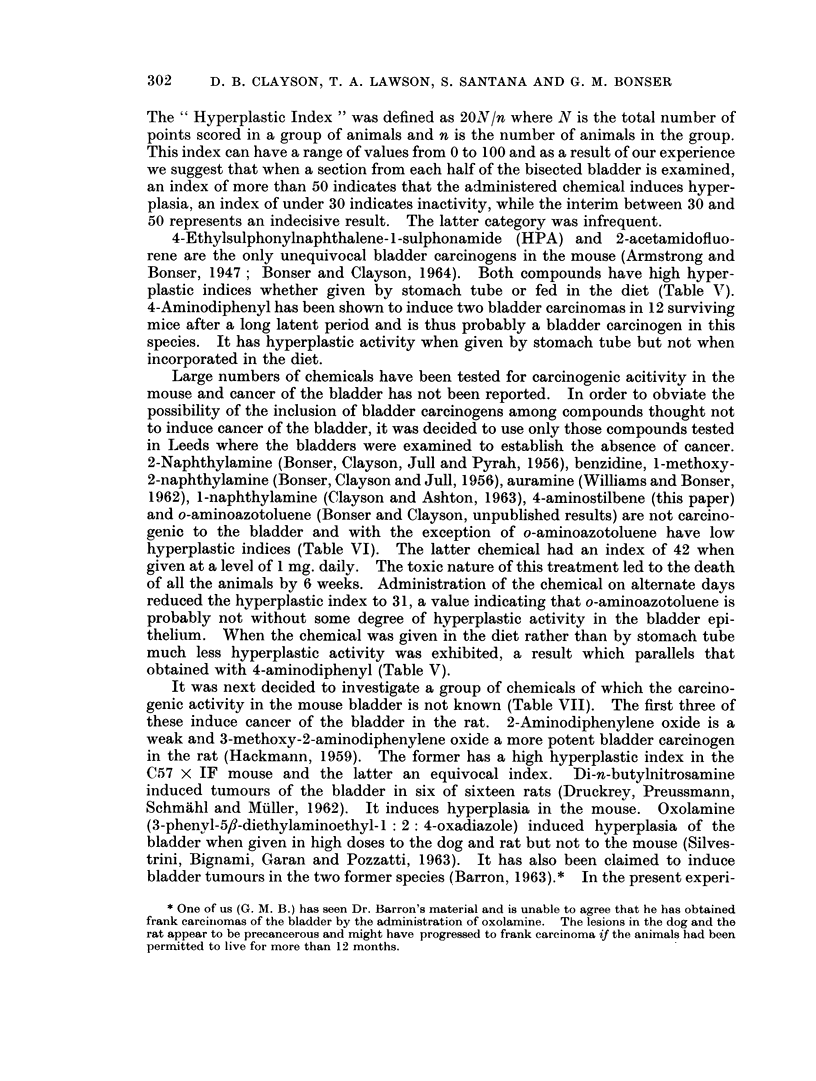

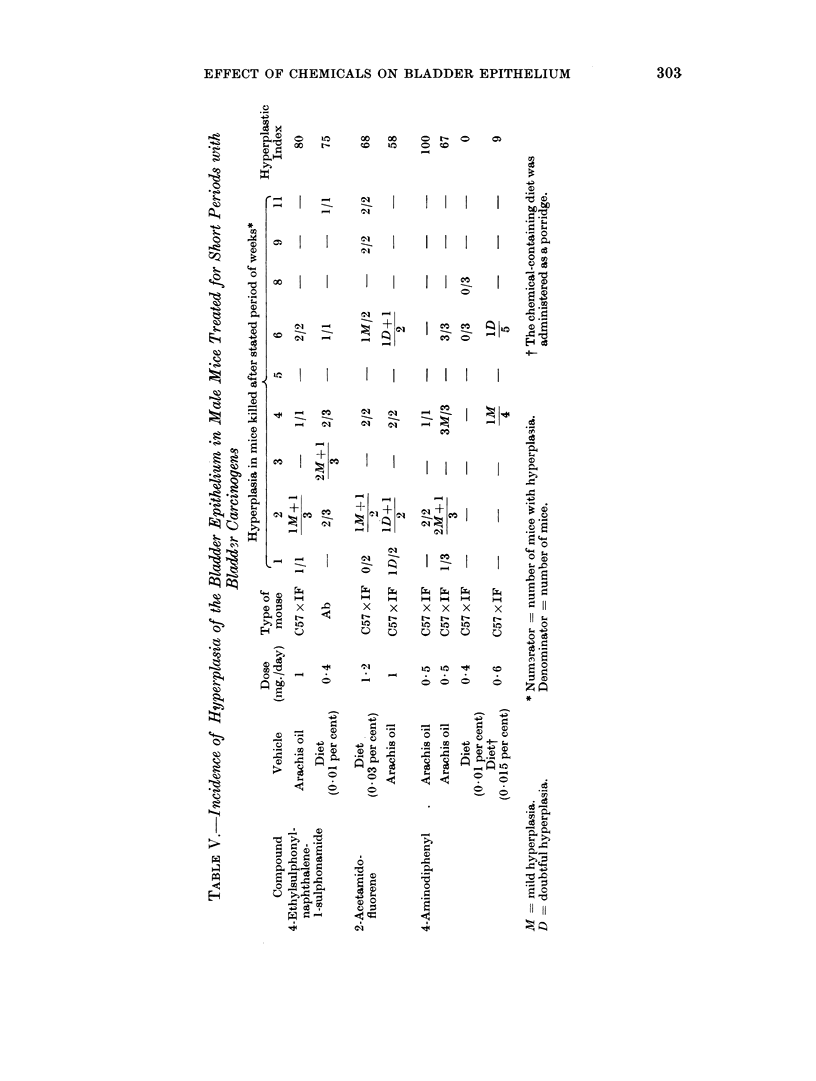

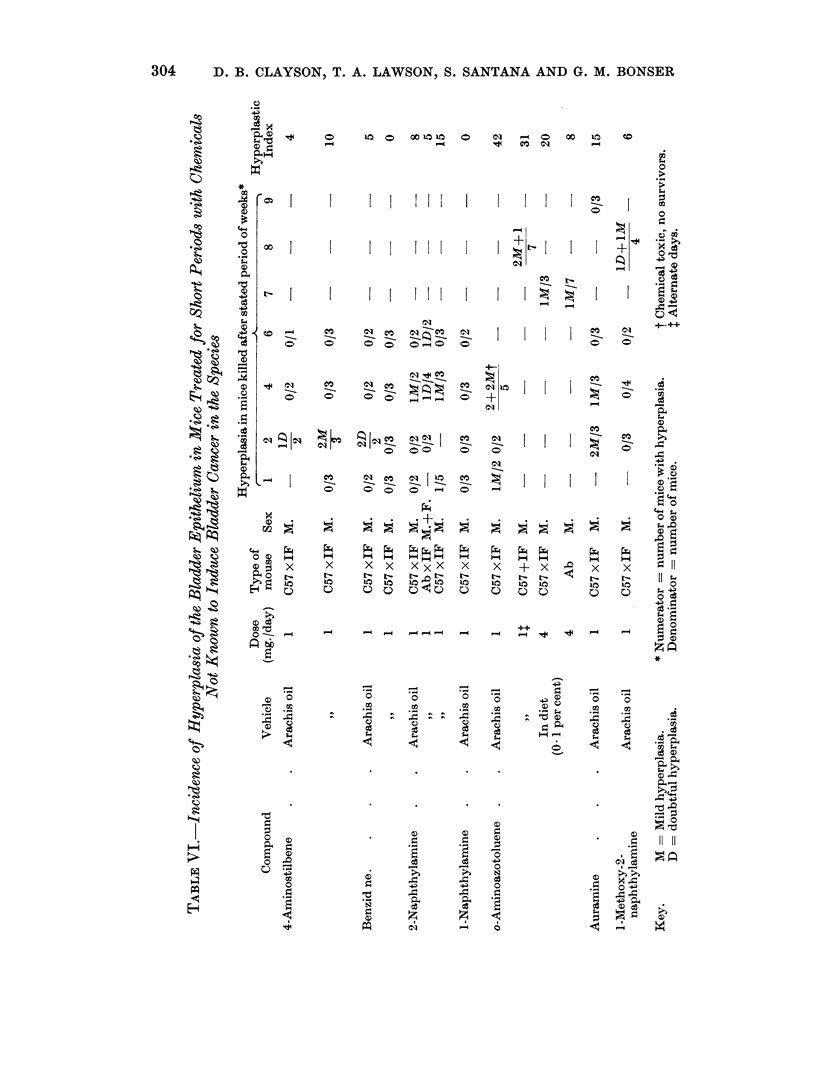

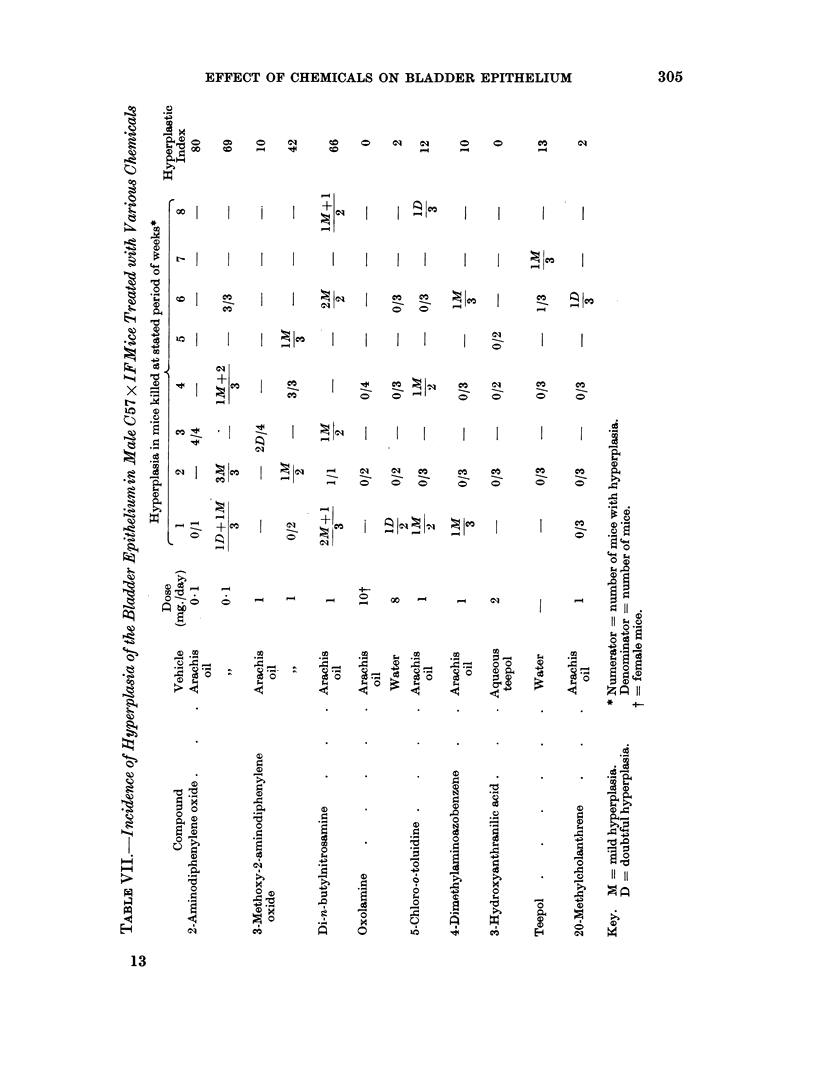

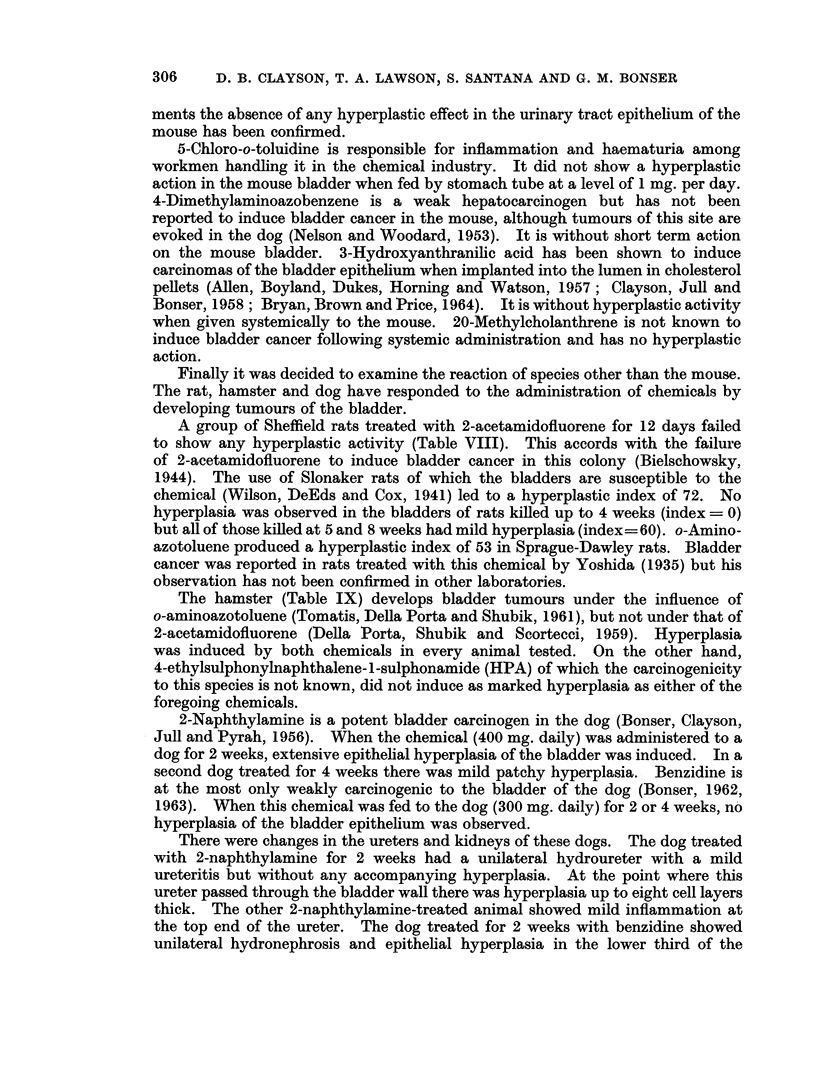

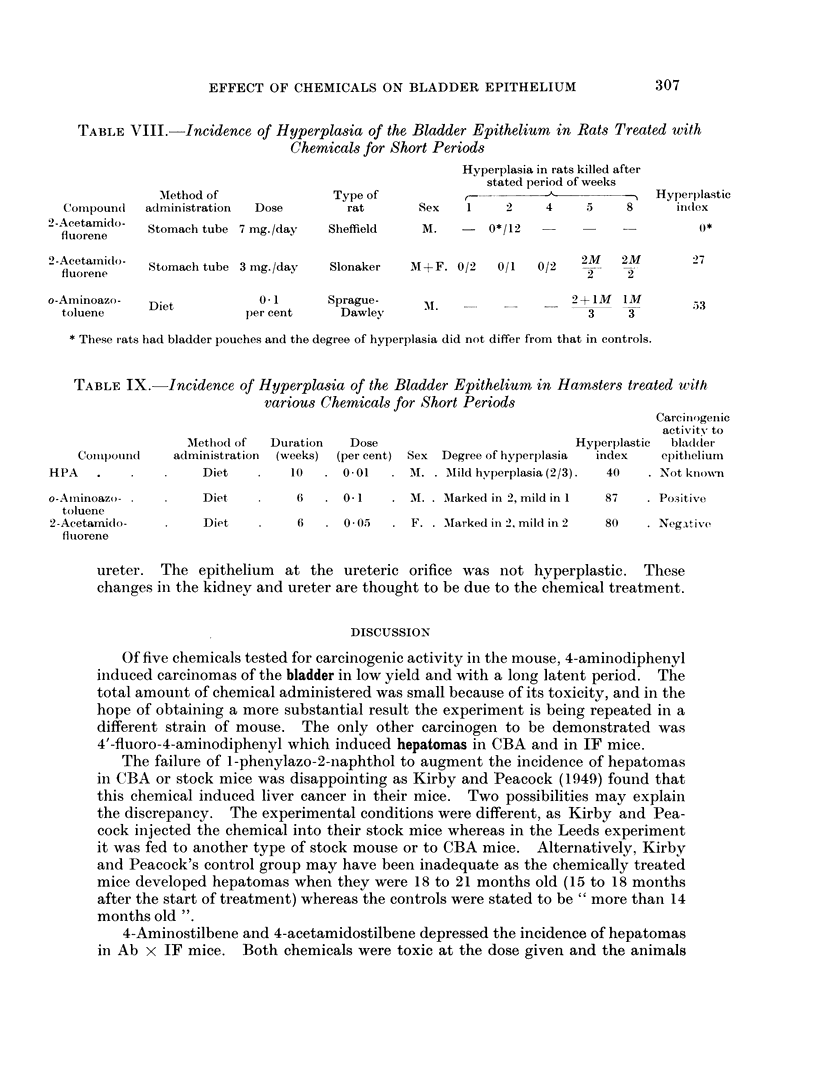

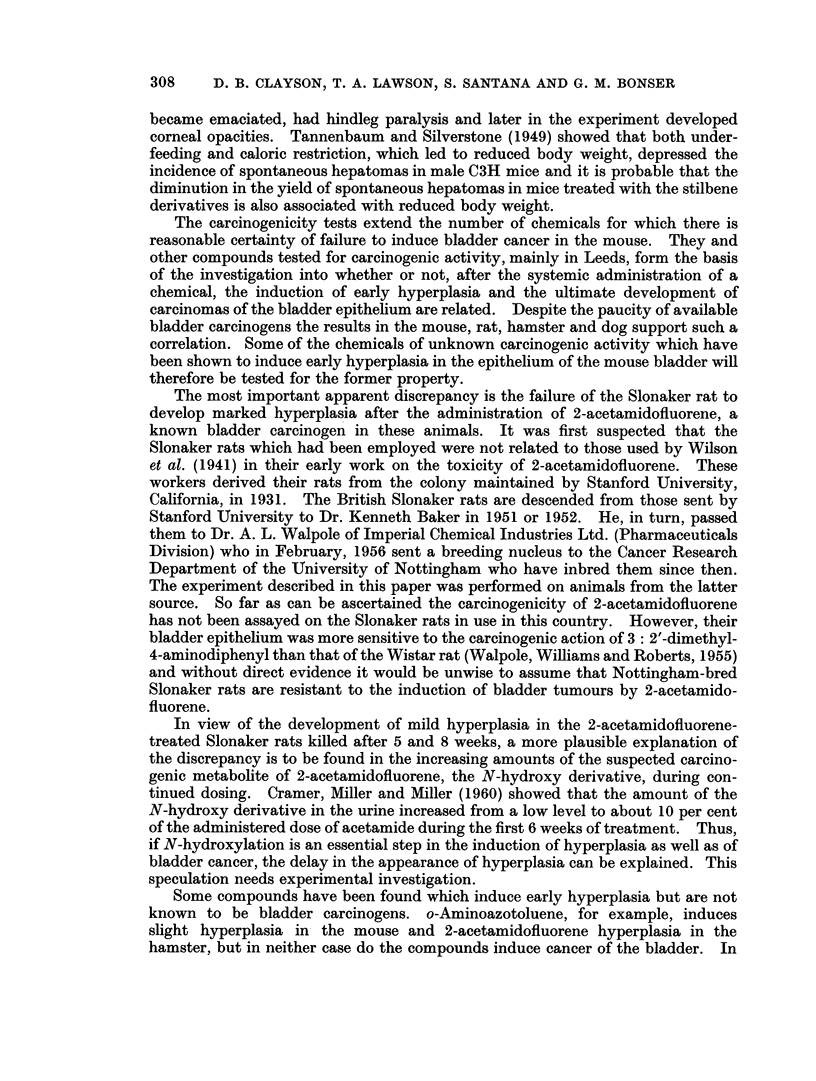

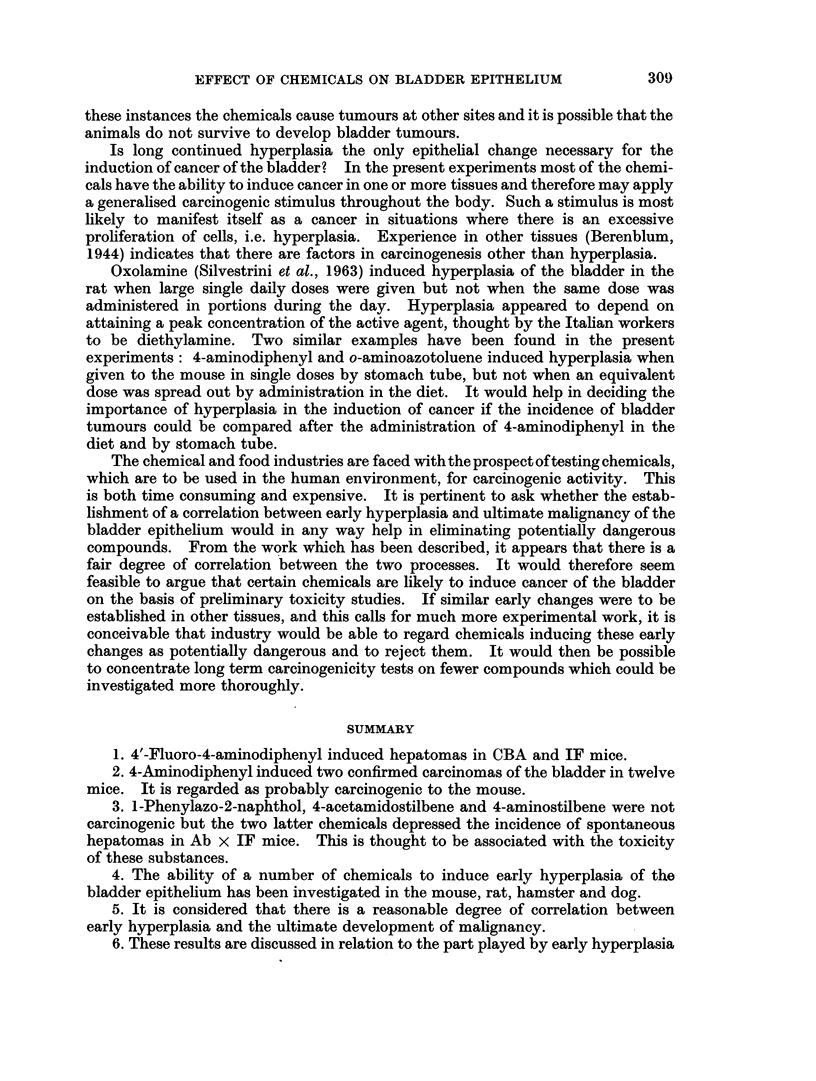

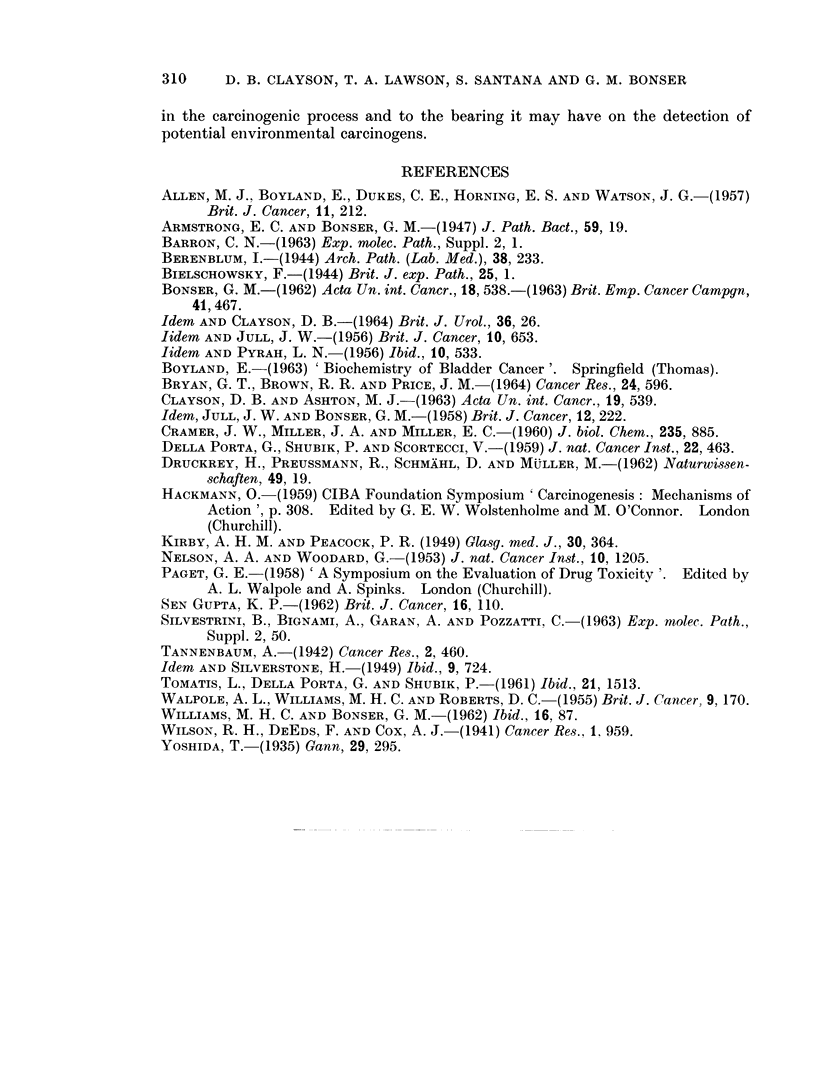

